# Evaluation of Nutrients Intake for a Group of Jordanian Older Adults with Sarcopenia Syndrome in Amman: An Explorative and Pilot Study

**DOI:** 10.1155/2021/6641967

**Published:** 2021-07-16

**Authors:** Sarah Ziad Al-Majali, Hadeel Ali Ghazzawi, Adam Tawfiq Amawi

**Affiliations:** ^1^Nutrition and Food Technology Department, School of Agriculture, University of Jordan, Amman, Jordan; ^2^Physical Education Department, School of Sport Science, University of Jordan, Amman, Jordan

## Abstract

**Aim:**

Sarcopenia is an age-related syndrome that is characterized by a progressive loss of muscle mass, strength, and function. This study was performed in order to evaluate nutrients intake and physical activity level and to investigate the effect of sarcopenia syndrome on food intake for a group of Jordanian older adults with sarcopenia syndrome in Amman. *Methodology*. The study sample consisted of 25 nonsarcopenic people and 25 sarcopenic patients aged over 60 years old with a male to female ratio of 1 : 1. A special questionnaire was used to collect demographic data, health data, data about syndrome characteristics, nutritional assessment, and physical activity level. A 24-hour recall was also used to collect food intake data. Body weight, height, and skinfold thicknesses were measured.

**Results:**

The mean of the sarcopenic patients' age was 77.5 ± 6.9 years, and the mean of the weight was significantly lower in sarcopenic patients than the nonsarcopenic people. In this study, all macronutrients and micronutrients from dietary intake information were analyzed. Vitamin intake (water- and fat-soluble), as well as minerals (major and trace), amino acids, and essential fatty acids, was assessed. The mean intake of energy and carbohydrates, fat, and dietary fiber was lower than their recommendations, while the mean intake of protein was within the range of its recommendations in the sarcopenia group. The mean of the intake of omega 3 and omega 6 was below their recommendations.

**Conclusion:**

It could be concluded that sarcopenic older patients in Jordan have similar characteristics with patients studied worldwide with regard to age of patients, female to male ratio, and main symptoms. Sarcopenic older patients in Jordan generally have lower weight and BF% than nonsarcopenic adults and have inadequate dietary intake compared to their recommendations and compared to nonsarcopenic older adults. Therefore, the diet of sarcopenic patients needs modification and follow-up. The level of physical activity and daily living activities for sarcopenic older patients is lower than that of nonsarcopenic older adults.

## 1. Introduction

Sarcopenia is a syndrome characterized by generalized and progressive loss of muscle strength, muscle mass, and integrity [1]. The adverse effects of sarcopenia syndrome include reduced ability in daily activities and increased risk of falling, and it plays an extremely important role within the etiology of frailty, higher morbidity, mobility disorders, and elderly mortality [2].

It appears that 5–13% of people aged from sixty to seventy years and 11–50% of people in their 80s have sarcopenia syndrome [3]. It is estimated that there are 3.6 million persons diagnosed with sarcopenia syndrome in the United States [4]. In the year 2000, healthcare costs attributed to sarcopenia syndrome in the United States were estimated to 18.5 billion dollars [5]. Yamada et al. [6] reported that the prevalence of sarcopenia syndrome in older people living in Japan was 21.8%, which is higher than the prevalence of older people in the United Kingdom (7.8%). World Health Organization (WHO) estimated that, in 2000, there were 600 million people aged 60 years or older, and this number may rise to 1.2 billion by 2025 [7].

Many factors lead to the development and progression of sarcopenia syndrome including muscular disuse, age-related alterations in sex hormones, protein synthesis, proteolysis, neuromuscular integrity, endocrine function, physical inactivity, and nutritional balance [8].

Sarcopenia syndrome may compromise adequate nutrition, if physical activity and performance are decreased. Shopping and cooking can also become burdensome and exhausting, the flexibility to arrange adequate meals is also reduced, and the appetite may be decreased as a result of low activity. In frail elderly, who need support from others for basic daily living activities, the risk of inadequate intake is further increased [9, 10]. In the pathogenesis of sarcopenia syndrome, malnutrition plays a key role, as both undernutrition and obesity increase the risk of sarcopenia syndrome in elderly people. The standard of the diet plays a key role within the incidence of sarcopenia syndrome; many nutritional interventions are also of interest in sarcopenia syndrome and frailty [11]. Poor dietary intake has been related to individuals of sarcopenia syndrome, possibly due to changes of dietary pattern, decreased response of aging muscle to anabolic stimuli from meals, or oxidative stress from comorbidities and aging [12].

Sarcopenia syndrome is a major concern for public health, and it is affected by many risk factors. In the complex etiology of the sarcopenia syndrome, nutrition is considered one of the most important contributing factors. Associations between several nutritional factors, muscle mass, strength, function, and physical performance were reported in a growing number of studies in recent years. Accordingly, while avoiding weight loss is crucial to prevent the concomitant loss of muscle mass, adequate amounts of high-quality protein are essential for optimal stimulation of muscle protein synthesis. Vitamin D, antioxidants, and omega 3-polyunsaturated fatty acids may also contribute to the preservation of muscle function [10].

To the best of our knowledge, no studies were done in Jordan to link the nutritional status and physical activity levels of sarcopenic patients. Therefore, the present study aims to evaluate food intake, physical activity level, and anthropometric measurements among a sample of older adults with sarcopenia syndrome, investigating the effect of sarcopenia syndrome on food intake among a selected sample of Jordanian older adults in Amman, comparing food intake recommendations of sarcopenic patients with their actual food intake and comparing food intake of sarcopenic patients with nonsarcopenic group.

## 2. Materials and Methods

### 2.1. Study Design

A cross-sectional study design was applied on a convenient sample of fifty Jordanian older adults aged more than 60 years in Amman during the period from November 2019 to February 2020. The sample consisted of two groups, a twenty-five older adults with sarcopenia syndrome compared to twenty-five nonsarcopenic older adults.

### 2.2. Study Sample

A total of 50 persons were assessed for eligibility to participate in this study. A total of 25 nonsarcopenic older males and females aged more than 60 years were recruited. Twenty-five older adults diagnosed with sarcopenia syndrome, males and females aged more than 60 years old, were recruited from the elderly care centers (*White beds* and *Darat Samir Shama*) and private clinics in geriatrics medicine (*Dr. Dana Abu-rub*). The inclusion criteria for including eligible older adults were male and female, age more than 60 years, diagnosis of sarcopenia syndrome by a physician, living in Jordan, Jordanian nationality, and physical ability. The exclusion criteria for excluding eligible older adults were as follows: age less than 60 years, not diagnosed with sarcopenia syndrome by a physician, and older adults with muscle wasting disease like cancer, type 1 diabetes mellitus (T1DM), protein energy malnutrition (PEM)), does not live in Jordan, not Jordanian, disabled, and Alzheimer disease.

### 2.3. Ethical Considerations

This study was approved by the committee of Graduate Studies and the Deanship of Academic Research and International Review Board (IRB) (decision number 138/2020/19). All participants were asked to sign a written consent. Before asking them for consents, all participants received verbal description of the study nature, objectives, and confidentiality. A written consent was obtained from all older adults who participated in the study. Names of the older adults of the sample were not included in the study in order to maintain full privacy and confidentiality.

### 2.4. Anthropometric Measurements

Height (*m*) and weight (kg) were measured for each participant by researchers according to the standard procedure as mentioned in [13] using a digital weight scale device (Beurer GS® 170) and digital stadiometer (InLab®). Each participant was asked to stand barefoot in the middle of the scale's base with the body weight (WT) equally distributed, and reading to the nearest 100 g (0.1 kg) was obtained. Height (HT) was measured as the participants were barefooted, stood straight with ankles together, and looked straight ahead with shoulders against the wall. Measurements to the nearest 0.1 cm were obtained [13].

Body mass index (BMI) was calculated according to the formula BMI = kg/m^2^, where kg is a person's weight in kilograms and m^2^ is their height in meters squared. The skinfold thickness (SFT) measurements were taken for the triceps muscle at the midpoint of the back of the upper left arm for the right-handed people and of the right hand for left-handed people using (Accu-measure® U.S) skinfold thickness caliper, which measures (SFT) to the nearest 0.2 mm, and with a maximum capacity of the caliper 60 mm. The Jackson and Pollock equation was used to calculate body density (*D*) in males and females (Table 1).

(1) $$\begin{array}{c} \text{Male}\left(D\right)=1.10938-\left(0.0008267\times \text{SFT in mm}\right)+\left(0.0000016\times \text{SFT in mm squared}\right)-\left(0.0002574\times \text{age}\right),\\ \text{Female}\left(D\right)=1.0994921-\left(0.0009929\times \text{SFT in mm}\right)+\left(0.0000023\times \text{SFT in mm squared}\right)-\left(0.0001392\times \text{age}\right). \end{array}$$

Body fat percentage (BF%) was calculated using the following equation [14]:

(2) $$\begin{array}{c} \text{BF}\%=\left[\left(\frac{4.95}{D}\right)-4.5\right]\times 100. \end{array}$$

### 2.5. Demographic and Health Data

Personal information (age, gender, and marital status divided into four groups: married, single, divorced, and widowed), questions about the health information (smoking, hookah, and chronic diseases), and questions about sarcopenic syndrome (duration of syndrome, management of the syndrome, and supplements used for it) were asked to the participants or caregivers during face to face interview and filled by the researchers.

### 2.6. Dietary Data

The nutritional risk was assessed according to the score of the nutritional assessment form used developed by [15–17] and validated by five professor specialists in human nutrition and dietetics at University of Jordan and University of Petra. The evaluation of nutrients intake was done by collecting food intake data using one day 24-hour recall. The participants or caregivers were asked to determine the quantity of the consumed food items by using household measures. Food intake of each subject was analyzed for energy, macronutrients, and micronutrients intake by using Food Processor SQL® (ESHA) software (Ver 11.1/2016). Measuring cups and food models were used to help participants estimate accurately their intake of food items.

### 2.7. Physical Activity Data

A 7-day physical activity recall was collected using physical activity form developed by [18] to measure the physical activity level among older adults according to the score of physical activity form, and the physical activity recall includes lower intensity activities, as well as moderate and high intensity activities, and was validated by five professor specialists in human nutrition and dietetics at University of Jordan and University of Petra. The collected physical activity level data were evaluated and analyzed using SPSS software.

### 2.8. Statistical Analysis

All data was collected and entered into the Statistical Program for Social Sciences Software® (SPSS for windows version (19) 2010, Chicago). Means and standard deviations were calculated for continuous variables, whereas categorical variables were reported as counts and frequency distribution (%). Differences between those in the sarcopenia group and nonsarcopenia group were estimated by using unpaired *t*-test for continuous variables and Chi-square for categorical variables.

The *P* value at 0.05 was set to assure statistical significance. The outcome data was presented in numbers, proportions, means, and standard deviations.

## 3. Results

### 3.1. Description of the Studied Sample

A total number of 50 older adults, 25 sarcopenic older adults and 25 nonsarcopenic older adults, were recruited in the study, with a 1 : 1 female to male ratio. The characteristics are presented in Table 2.

### 3.2. Anthropometric Measurements

The anthropometric measurements (weight, height, BMI, and body fat percentage) are presented in Table 3. This table shows that mean weight was significantly lower in sarcopenia group compared to nonsarcopenia group. The mean height was not significantly different between the sarcopenia group and nonsarcopenia group. According to the classifications of BMI, the normal BMI category mean was the highest followed by underweight, and the obese category was the lowest among sarcopenia group. However, the normal BMI mean was the highest followed by overweight, while the obese and underweight categories means were the lowest among the nonsarcopenia group. The BMI was almost significant between the two groups, lower in sarcopenia group compared to nonsarcopenia group. According to the BF% categories, the BF% was the highest at lean category and lower at above average category among sarcopenia group, whereas the BF% was highest at average category and lower at lean category among nonsarcopenia group. The BF% was significantly lower in sarcopenia group compared to nonsarcopenia group.

### 3.3. Health Information

The health information (smoking, hookah, and chronic diseases) are presented in Table 4. This table shows that the smoking and hookah behaviors did not significantly differ between the sarcopenia group and nonsarcopenia group. Also, presence of chronic diseases is presented in Table 5 and it was not significantly different between the two groups. Chronic diseases (divided into categories: osteoporosis disease and hypertension) were the highest among sarcopenia group. Hypertension was the highest in the nonsarcopenia group. The category of disease was highly significant between the two groups.

### 3.4. Syndrome Characteristics

**Figure 1:**
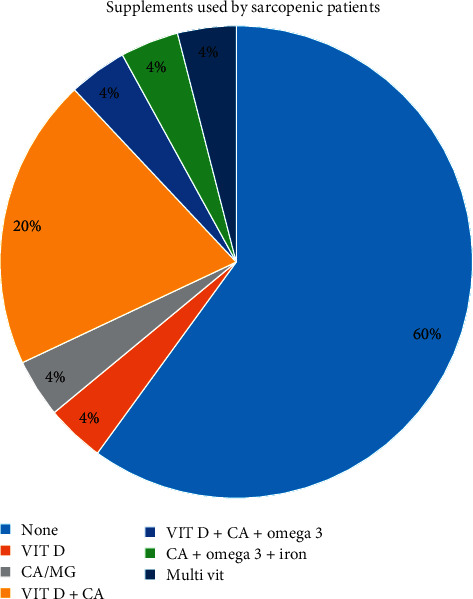
The main types of supplements used by the sarcopenic patients included in the study.

**Figure 2:**
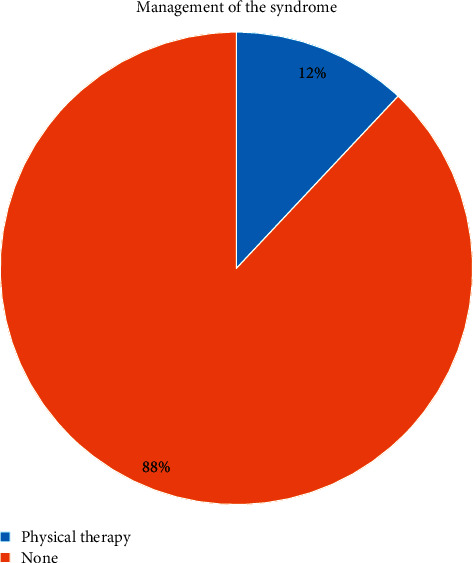
Pie chart for the management of sarcopenia syndrome by the sarcopenic patients included in the study.

Syndrome characteristics among sarcopenia group refer to the duration of syndrome, management of syndrome, and supplements used. Mean value of sarcopenia syndrome patients' duration in years is (2.5 ± 1.54). The major supplements used by sarcopenic patients are presented in Figure 1. The most consumed supplement by sarcopenic patients was vitamin D + calcium. Management of sarcopenia syndrome is presented in Figure 2.

### 3.5. Energy and Macronutrients Intakes

The mean intake of energy, fiber, and macronutrients of the sarcopenic patients and nonsarcopenic people included in the study is shown in Table 6. This table shows that the mean intake of calories, food weight in grams, protein, carbohydrates, fat, and total dietary fiber was significantly lower in sarcopenia group compared to nonsarcopenia group.

The mean intake of energy and macronutrients and their recommendations are shown in Table 7. The mean intake of calories, carbohydrates, fat, and dietary fiber was lower than their recommendation. The mean intake of energy was significantly lower in sarcopenic patients compared to their recommendations. However, the mean intake of protein was within the range of its recommendation and did not show any significant difference between sarcopenic patients' intake and their recommendation.

#### 3.5.1. Fatty Acids and Cholesterol

The mean intake of fatty acids and cholesterol of the sarcopenic patients and nonsarcopenic people included in the study is shown in Table 8. This table shows that the mean intake of saturated fat was significantly lower in sarcopenia group compared to nonsarcopenia group. Also, the mean intake of monounsaturated fatty acid (MUFA) was significantly lower in sarcopenia group compared to nonsarcopenia group. The mean intake of polyunsaturated fatty acid (PUFA) did not significantly differ between the sarcopenia group and nonsarcopenia group. The mean intake of trans fat was not significantly different between the sarcopenia group and nonsarcopenia group. The mean intake of cholesterol was significantly lower in sarcopenia group compared to nonsarcopenia group. The mean intake of omega 3 was not significantly different between the sarcopenia group and nonsarcopenia group. The mean intake of omega 6 was not significantly different between the sarcopenia group and nonsarcopenia group.

The mean intake of fatty acids and cholesterol and their recommendations is shown in Table 9. The mean intake of SFA, PUFA, MUFA, cholesterol, omega 3, and omega 6 was lower than their recommendations. The mean intake was significantly lower in sarcopenic patients compared to their recommendations.

#### 3.5.2. Amino Acids

The mean intake of amino acids of the sarcopenic patients and nonsarcopenic people included in the study is shown in Table 10. This table shows that there were significant differences between the intake of histidine, isoleucine, leucine, threonine, and tryptophan between the sarcopenia group and nonsarcopenia group; sarcopenia group was lower than nonsarcopenia group. There were no significance differences in the intake of lysine, methionine + cysteine, phenylalanine, and valine between sarcopenia group and nonsarcopenia group.

### 3.6. Micronutrients Intake

#### 3.6.1. Fat-Soluble Vitamins

The mean intake of fat-soluble vitamins (A, D, E, and K) of the sarcopenic patients and nonsarcopenic people included in the study is shown in Table 11. This table shows that the mean intake of fat-soluble vitamins was not significantly different between the sarcopenia group and nonsarcopenia group.

The mean intake of fat-soluble vitamins and their recommendations is shown in Table 12. The mean intake of vitamin A, vitamin D, and vitamin E was below to their recommendation. The mean intake was significantly lower in sarcopenic patients compared to their recommendations. However, the mean intake of vitamin K was higher than the recommendation and showed no significant difference between sarcopenic patients intake and their recommendations.

#### 3.6.2. Water-Soluble Vitamins

The mean intake of water-soluble vitamins of the sarcopenic patients and nonsarcopenic people included in the study is shown in Table 13. This table shows that the mean intake of all B-vitamins was not significantly different between the sarcopenia group and nonsarcopenia group. However, the mean intake of vitamin C was significantly lower in sarcopenia group compared to nonsarcopenia group.

The mean intake of water-soluble vitamins and their recommendations is shown in Table 14. The mean intake of vitamin B_1_, vitamin B_3_, vitamin B_6_, biotin, folate, and pantothenic acid was below than their recommendations. The mean intake was significantly lower in sarcopenic patients compared to their recommendations. However, the mean intake of vitamin B_2_, B_3_ niacin equivalent, and vitamin C was within their recommendations and showed no significant difference between sarcopenic patients intake and their recommendation. The mean intake of vitamin B_12_ was higher than its recommendations with close significance.

#### 3.6.3. Major and Trace Minerals

The mean intake of minerals of the sarcopenic patients and nonsarcopenic people included in the study is shown in Table 15. This table shows there were significant differences between the intakes of copper, fluoride, iodine, magnesium, manganese, and potassium between the sarcopenia group and nonsarcopenia group; sarcopenia group was lower than nonsarcopenia group. However, there was no significant difference in the intake of calcium, chromium, iron, molybdenum, phosphorus, selenium, sodium, and zinc between sarcopenia group and nonsarcopenia group.

The mean intake of minerals and their recommendations is shown in Table 16. The mean intake of calcium, chromium, copper, fluoride, iodine, magnesium, molybdenum, potassium, and zinc was lower than their recommendations. The mean intake was significantly lower in sarcopenic patients compared to their recommendations. However, the mean intake of selenium, manganese, phosphorous, iron, and sodium showed no significance difference between sarcopenic patients intake and their recommendations.

### 3.7. Nutritional Assessment

The nutritional assessment for sarcopenic patients compared to nonsarcopenic people is presented in Table 17. This table shows that the daily living activities, eating without help, preparing meals, shopping for food, picking up of the food, grasping of utensils and cup, getting food from utensils, and bringing food and cup to mouth, were significantly lower in sarcopenic patients than the nonsarcopenic people.

There were significant differences between sarcopenic patients and nonsarcopenic people in chewing and swallowing foods and liquids, cleaning mouth and hands, weight loss during the last 3 months, and decline in food intake (*P* < 0.050). Also, there was a significant difference between sarcopenic patients and nonsarcopenic people in number of full meals. However, there is no significant difference in snacks.

There were significant differences between sarcopenic patients and nonsarcopenic people in major food groups consumed per day: vegetables, fruit, water, tea and coffee, rice, meat and chicken, egg, and nuts. However, there were no significant differences in the consumption in dairy products, milk and juice, while bread and rice intake was close to significance.

The nutritional risk assessment for the sarcopenic patients and nonsarcopenic people included in the study is shown in Table 18. This table shows that the nutritional risk was significantly higher in sarcopenic patients compared to nonsarcopenic people according to the score of nutritional assessment form. The classification of nutritional risk was high, moderate, and none. The nutritional risk was the highest at moderate nutritional risk and lower at highly nutritional risk among sarcopenia group, while “No nutritional risk” category was the highest and lower at high nutritional risk among nonsarcopenia group.

### 3.8. Physical Activity

The physical activity for sarcopenic patients compared with nonsarcopenic people is presented in Table 19. This table shows that walking outside home, doing light sports, doing exercise to increase muscle strength, light house work, and heavy housework were significantly different between sarcopenic patients and nonsarcopenic people. However, there are no significant differences in moderate sports and extraneous sports between sarcopenic patients and nonsarcopenic people.

The physical activity level for the sarcopenic patients and nonsarcopenic people included in the study is shown in Table 20. This table shows that the physical activity level was significantly lower in sarcopenic patients compared to nonsarcopenic people. According to the score of physical activity level, the classifications of physical activity levels were high, moderate, light, and sedentary. The physical activity level was the highest at sedentary activity category, followed by lightly active category, and lower at moderately and highly active categories among sarcopenia group. The lightly active category was the highest followed by moderately active category and lower at highly active category among nonsarcopenia group.

## 4. Discussion

Sarcopenia is an age-related syndrome that is associated with many nutritional disturbances. This study aims to evaluate the nutritional status comparison of sarcopenic patients and nonsarcopenic people in Jordan, through assessing their nutrient intake, physical activity level, and measuring some anthropometric indices.

The importance of this study may be summarized in these points; first, the increasing prevalence of sarcopenia syndrome in Jordan and worldwide and without diagnosis in Jordan due to lack of interest; second, the accumulating evidence on the efficacy of dietary supplements and the use of certain diets for the alleviation of its symptoms; and third, the general lack of nutritional assessment of patients, particularly, in Jordan. In this study, evaluation has been made for sarcopenic patients through anthropometric measurements, physical activity level, and nutritional intake. To the best of our knowledge, no studies have been done in Jordan to evaluate the nutritional status of sarcopenic patients.

It should be stated that it was extremely difficult to recruit subjects and obtain the study sample due to the small number of patients diagnosed with this syndrome and obtain data from them because of the consideration of the exclusion criteria and of the psychological conditions and mood disturbances that the sarcopenic patients have.

Food recalls were used to assess the macronutrients, micronutrient intake, essential fatty acid, and amino acids among 50 older adults people in Amman, Jordan, and were analyzed using Food Processor SQL® software. Generally, the consumption of nutrients in this study was found to be relatively low among the sarcopenic patients.

The syndrome most commonly affects people more than 60 years old; the mean age of sarcopenic patients in this sample was 77.5 years old, which is extremely close to the mean age (74.8 and 76.05 year old) in the studies conducted by [19]; [1] respectively).

Female to male ratio in our study was almost 1 : 1, which is extremely close to the ratio (1 : 1) in the study by [12]. Generally, males are more exposed to sarcopenia syndrome possibly due to the fact that males tend to lose muscle mass gradually with age, but the decline in muscle mass with age is insignificant or slightly significant in females [20].

Regarding the anthropometric assessment of the participants, mean body weight of the participants in this study was significantly lower in sarcopenic patients compared to nonsarcopenic people (59.44 ± 11.7 and 66.7 ± 5.6 kg, respectively*P*=0.007). [21] found that the weight was significantly lower in sarcopenia group than nonsarcopenia group (*P*=0.002). However, BMI is compared, and it was found that the BMI differences ended to be significant between sarcopenic patients and nonsarcopenic people (*P*=0.057). Most sarcopenic patients included in this study (68%) were in the normal BMI class, followed by underweight class (20%) and the least percentage in obese class (4%). These findings support what was found by [1, 21, 22]; i.e., BMI was significantly lower in sarcopenia group compared to nonsarcopenia group (*P*=0.048).

Results also show that BF% was within lean category for 60% (more than half) of the sarcopenic patients. This may be attributed to the nature of the syndrome and the difficulties in eating. Also, it is clear from the results that nonsarcopenic participants have significantly higher body fat percent than sarcopenic patients, which is consistent with what is true for any general population.

Sixty percent of sarcopenic patients included in the present study were not using dietary supplements. It is documented in the literature that sarcopenic patients should use a supplement. The use of supplements may alleviate the nutrient deficiencies associated with the syndrome. [21] found that sarcopenic older adults differed in certain nutritional intake and biochemical nutrient status compared with nonsarcopenic older adults. Dietary supplement intake reduced the gap for some of these nutrients. Targeted nutritional intervention may, therefore, improve the nutritional intake and biochemical status of sarcopenic older adults.

In 2017, a study was conducted by Verlaan et al. to compare functional and nutritional status, body composition, and quality of life of older adults between age and sex-matched older adults with and without sarcopenia. Sarcopenic participants reported significantly less physical activity level than the nonsarcopenic adults and significantly lower ability to perform daily activities. Sarcopenic older adults reported having a poorer health-related quality of life than the nonsarcopenic adults (*P* < 0.001). For similar energy intake (sarcopenia group 1710 ± 418; nonsarcopenia group 1745 ± 513, *P*=0.50), the sarcopenia group consumed less protein, vitamin D, vitamin B_12_, magnesium, phosphorus, and selenium compared to nonsarcopenia group (with *P* < 0.05). The serum concentration of vitamin B_12_ was lower in the sarcopenia group (*P*=0.015). These findings are in agreement with the findings of our study in protein intake, magnesium intake, physical activity level, and poorer health-related quality of life. It was concluded that the lower micronutrient density of the sarcopenic group's diets and the nutrient intake were significantly lower and could signal a lower quality of the diet in the sarcopenic group. Thus, a group of nutrients rather than individual nutrients could also contribute to lower muscle mass, strength, and function of sarcopenia syndrome. [23] showed that higher intake of calcium, magnesium, niacin, phosphorus, potassium, riboflavin, and zinc had positive increasing trends for increased appendicular muscle mass.

In 2019, a study was conducted by Beaudart to describe cross-sectional associations between dietary nutrient intake and sarcopenia syndrome. A total of 331 subjects (mean age of 74.8 ± 5.9 years, 58.9% women) had complete data and were included in the study. Sarcopenic subjects were older, with a lower BMI. They also presented lower strength, lower muscle mass, and lower physical activity test scores. Analyses revealed that sarcopenic subjects consumed significantly lower amounts of two macronutrients (proteins, lipids) and five micronutrients (potassium, magnesium, phosphorus, iron, and vitamin K) than nonsarcopenic subjects (all *P* values < 0.005). It was concluded that sarcopenic subjects seem to consume significantly reduced amounts of micronutrients and macronutrients compared to nonsarcopenic subjects. These results suggest that a poorly balanced diet may be associated with sarcopenia syndrome and poor musculoskeletal health. Findings of our study are in agreement with these findings.

Proteins represent the most important macronutrient in counteracting muscle mass loss in the elderly. Indeed, protein provides the essential amino acids that are needed for the synthesis of muscle protein and acts as an anabolic stimulus with direct effects on protein synthesis. Not surprisingly in this research, sarcopenic subjects consumed significantly less protein than nonsarcopenic subjects.

Comparable results were also observed in a study similar to this one, performed in Maastricht, which found that nonsarcopenic subjects consumed approximately 74 ± 20 g of protein/day, while sarcopenic subjects consumed 68 ± 22 g of protein/day (*P*=0.048) [21]. The amount of protein intake is important to maximize the anabolic response. Approximately 35% of total calories should be composed of proteins, and regarding the amino acid composition of proteins, it has been suggested that proteins rich in amino acid (leucine) seem to be the best proteins in terms of anabolic properties [24]. These findings are extremely close to our results, where the mean intake of protein and amino acid leucine is significantly lower in sarcopenia group compared to nonsarcopenia group (*P* < 0.001 and 0.026, respectively).

A systematic review was done by [25] to evaluate the role of calcium, iron, magnesium, phosphorus, potassium, selenium, sodium, and zinc on muscle mass, muscle strength, and physical performance in older adults. From the 3346 articles found, a total of 10 studies met the inclusion criteria. Observational studies showed that serum selenium and calcium intake were significantly associated with muscle mass, and magnesium, selenium, iron, and zinc intake was significantly and positively associated with physical performance in older adults. Furthermore, magnesium, selenium, calcium, and phosphorus intake was associated with the prevalence of sarcopenia syndrome. Magnesium supplementation improved physical performance based on one-randomized-controlled trials. Our study shows that the mean intake of calcium, chromium, copper, fluoride, iodine, magnesium, molybdenum, potassium, and zinc was below their recommendations. The mean intake was significantly lower in sarcopenic patients compared to their recommendations (*P* < 0.050). It was concluded that minerals may be important nutrients to prevent and/or treat sarcopenia syndrome. Particularly, magnesium, selenium, and calcium seem to be more promising.

A prospective study was conducted by [26] to investigate the association between malnutrition sarcopenia syndrome and long-term mortality in older inpatients. Muscle mass and malnutrition were estimated by anthropometric measures and the Mini Nutritional Assessment. In 453 participants, the mean age of the study participants was 79 years old, compared with nonsarcopenic participants, and sarcopenic participants were more prone to have malnutrition and lower BMI, compared with normally nourished participants without sarcopenia syndrome. Our results show that the nutritional risk was significantly higher in sarcopenic older adults compared to their controls (*P* < 0.001). The nutritional risk was the highest at “moderate nutritional risk” category (68%, *N* = 17) and lower at “highly nutritional risk (8%, *N* = 2) among sarcopenic group. “No nutritional risk” category was the highest (96%, *N* = 24) and lower at high nutritional risk (4%, *N* = 1) among nonsarcopenia group. These findings are in agreement with the findings of a study conducted in 2020 by Xu et al. to investigate the association between sarcopenia syndrome and disability in activities of daily living and physical function among the oldest old. These authors found that older age, lower BMI, and worse nutritional status were significantly associated with the presence of sarcopenia syndrome. Sarcopenia syndrome was independently associated with disability and poor physical function. Our results show that the activities of daily living, eating without help, preparing meals, shopping for food, picking up the food, grasping utensils and cup, getting food from utensils, and bringing food and cup to mouth, were significantly lower in sarcopenic patients than the nonsarcopenic people (*P* < 0.050).

The strength of this study is that there were no studies done in Jordan to find the association of nutritional status of sarcopenic patients with the syndrome. Therefore, research in this topic will be the beginning for a lot of future research. However, the study drew a picture describing the macro- and micronutrients intake, nutritional risk assessment, physical activity level, and anthropometric measurements in a group of sarcopenic patients living in Amman. Although the study has good important findings, it also has some limitations: first, the relatively small sample size. Second, although the quantity of food items recalled by patients is determined according to given instructions and food models, it could be over- or underestimated. Third, the study lacks biochemical nutrient assessment to confirm the nutritional status. Fourth, we use one-day 24 hr recall.

## 5. Conclusions, Recommendations, and Limitation

### 5.1. Conclusions

The results of this study showed that sarcopenic patients in Jordan have similar characteristics with patients studied worldwide regarding age of patients and female to male ratio. Sarcopenic older adults patients in Jordan generally have lower weight and BF% than nonsarcopenic older adults, inadequate dietary intake for sarcopenic older adults patients compared to their recommendations and nonsarcopenic older adults, since only few nutrients were found to be adequately taken by them; and therefore, activating the role of a nutritionist is important in order to counsel sarcopenic older adults patients to improve their nutritional intake.

The diet of the sarcopenic older patients needs modification and follow-up, because the nutritional risk was the highest at “moderate nutritional risk” category (68%, *N* = 17). It was found that the supplements consumption goal, in order to increase micronutrient intakes level, was not consumed by sarcopenic older patients; only 40% sarcopenic older patients consumed minerals and vitamins supplements. It was also found that only 12% sarcopenic older patients manage the syndrome with physical therapy. In addition, the level of physical activity and daily living activities for sarcopenic older patients is lower than nonsarcopenic older adults.

### 5.2. Recommendations

Dietary management for sarcopenic older patients should start at diagnosis with assessment of nutritional status and nutritional counseling. The use of dietary supplements is recommended after consulting a nutritionist, especially for the deficient nutrients in the diet of the sarcopenic older patients. Long-term dietary intervention should be applied with careful monitoring of the nutritional status of sarcopenic older patients to prevent complications of nutrient deficiencies that may worsen the syndrome and its symptoms. There should be educational programs for the sarcopenic older patients, nonsarcopenic older adults, and their families in order to improve overall nutrition. Further research should be carried out to investigate sarcopenia syndrome prevalence in Jordan.

### 5.3. Limitation

Although the study has good important findings, it also has some limitations: first, the relatively small sample size. Second, although the quantity of food items recalled by patients is determined according to given instructions and food models, it could be over- or underestimated. Third, the study lacks biochemical nutrient assessment to confirm the nutritional status. Fourth, we use one-day 24 hr recall instead of food records (Table 21).

## Figures and Tables

**Table 1:** BMI and BF% cutoffs for identifying weight status.

Weight status	BMI	Body fat status	Fat (%)	
			Male	Female
Underweight	<18.5	Lean	9.9–19	16.3–26.4
Normal	18.5–24.9	Ideal	19.1–26	26.5–31.2
Over weight	25.0–29.9	Average	26.1–30.8	31.3–37.2
Obese	≥30.0	Above average	30.9–33.3	37.3–39.5

(WHO, 1995) (Accu-measure® U.S).

**Table 2:** General characteristics of the study sample.

Variables	Sarcopenia group *N* (%)	Control group *N* (%)	*P* value
Age (years)	77.5 ± 6.9	74.7 ± 5.5	0.070

*Gender*			
Female	13 (52)	13 (52)	
Male	12 (48)	12 (48)	

*Marital status*			
Married	5 (20)	19 (76)	≤0.001^*∗*^
Single	8 (32)	2 (8.0)	
Divorced	1 (4.0)	2 (8.0)	
Widowed	11 (44)	2 (8.0)	

Age is presented as mean age ± SD, *N* represents the number of participants, and (%) represents the percentage of the participants.

**Table 3:** Anthropometric measurements of the study sample.

Variables	Sarcopenia group *N* (%)	Nonsarcopenia group *N* (%)	*P* value
Weight (kg)	59.44 ± 11.7	66.7 ± 5.6	0.007^*∗*^
Height (m)	1.63 ± 0.10	1.68 ± 0.07	0.48

*BMI (kg/m* ^*2*^)			
Underweight	5 (20)	0 (0.0)	
Normal	17 (68)	24 (96)	0.057
Overweight	2 (8.0)	1 (4)	
Obese	1 (4.0)	0 (0.0)	

*Body fat (%)*			
Lean	15 (60)	0 (0.0)	
Ideal	5 (20)	4 (16.0)	≤0.001^*∗*^
Average	4 (16)	20 (80.0)	
Above average	1 (4.0)	1 (4.0)	

Weight and height are presented as mean ± SD, *N* represents the number of participants, (%) represents the percentage of the participants, and ^*∗*^*P* ≤ 0.05is set as significant.

**Table 4:** Health information for the participants.

Variables	Sarcopenia group *N* (%)	Non-sarcopenia group *N* (%)	*P* value
*Cigarette smoking*			
Yes	7 (28)	7 (28)	—
No	18 (72)	18 (72)	

*Hookah*			
Yes	0 (0)	0 (0)	—
No	25 (100)	25 (100)	

*Chronic diseases*			
Yes	15 (60)	9 (36)	0.089
No	10 (40)	16 (64)	

**Table 5:** Categories of chronic diseases for the participants.

Category of chronic diseases	Sarcopenia group no. (%)	Nonsarcopenia group no. (%)	*P* value
Cardiovascular disease	2 (8)	0 (0)	≤0.001^*∗*^
Hypertension	5 (20)	8 (88.9)	
Cardiovascular disease and hypertension	0 (0)	1 (11.1)	
Diabetes and hypertension	2 (8)	0 (0)	
Osteoporosis and hypertension	4 (16)	0 (0)	
Osteoporosis and cardiovascular disease	1 (4)	0 (0)	
Osteoporosis	1 (4)	0 (0)	

*N* represents the number of participants, (%) represents the percentage of the participants, and ^*∗*^*P* ≤ 0.05 is set as significant.

**Table 6:** Mean intake of energy, fiber, and macronutrients for the participants included in the study.

Macronutrients	Sarcopenia group mean ± SD	Nonsarcopenia group mean ± SD	*P* value
Weight (gram)	1135.3 ± 251.0	1923 ± 336.5	≤0.001^*∗*^
Energy	1082.7 ± 283.2	1705.6 ± 503.8	≤0.001^*∗*^
Protein (g)	47.5 ± 14.2	71.3 ± 17.8	≤0.001^*∗*^
CHO (g)	137.3 ± 49.6	204.7 ± 71.2	≤0.001^*∗*^
Total dietary fiber (g)	10.4 ± 5.7	22.3 ± 8.8	≤0.001^*∗*^
Fat (g)	39.9 ± 15.9	70.7 ± 31.7	≤0.001^*∗*^

^*∗*^ *P* ≤ 0.05 is set as significant, SD: standard deviation, CHO: carbohydrate, and (g): gram.

**Table 7:** Mean intake of energy and macronutrients for the sarcopenic patients compared to their recommendations.

Macronutrients	Intake mean ± SD	RCMD mean ± SD	*P* value
Calories (Kcal)	1082.8 ± 283.2	1693.2 ± 270.4	≤0.001^*∗*^
CHO (g)	137.3 ± 49.5	232.8 ± 37.2	≤0.001^*∗*^
Protein (g)	47.5 ± 14.1	47.5 ± 9.4	0.982
Fat (g)	39.9 ± 15.9	52.7 ± 8.4	0.001^*∗*^
Total dietary fiber (g)	10.4 ± 5.7	23.7 ± 3.8	≤0.001^*∗*^

^*∗*^ *P* ≤ 0.05 is set as significant, SD: standard deviation, RCMD: recommendation, CHO: carbohydrate, and (g): gram.

**Table 8:** Mean intake of fatty acids and cholesterol for the participants included in the study.

Variables	Sarcopenia group mean ± SD	Nonsarcopenia group mean ± SD	*P* value
Saturated fat (mg)	11.7 ± 4.4	15.9 ± 6.0	≤0.001^*∗*^
MUFA (mg)	10.6 ± 5.8	26.7 ± 16.9	≤0.001^*∗*^
PUFA (mg)	9.2 ± 8.1	10.6 ± 8.8	0.536
Trans fat (mg)	0.18 ± 0.16	0.11 ± 0.21	0.143
Cholesterol (mg)	151.4 ± 79.1	245.8 ± 136.9	0.004^*∗*^
Omega 3 (mg)	0.39 ± 0.2	0.48 ± 0.5	0.403
Omega 6 (mg)	2.10 ± 1.9	3.50 ± 3.7	0.103

^*∗*^ *P* ≤ 0.05 is set as significant, SD: standard deviation, and (mg): milligram.

**Table 9:** Mean intake of fatty acids and cholesterol for the sarcopenic patients compared to their recommendations.

Fatty acids	Intake mean ± SD	RCMD mean ± SD	*P* value
SFA (mg)	11.7 ± 4.4	16.9 ± 2.7	≤0.001^*∗*^
PUFA (mg)	9.2 ± 8.0	16.9 ± 2.7	≤0.001^*∗*^
MUFA (mg)	10.5 ± 5.8	18.8 ± 3.0	≤0.001^*∗*^
Cholesterol (mg)	151.3 ± 79.1	300 ± 0.0	≤0.001^*∗*^
Omega 3 (mg)	0.39 ± 0.2	1.69 ± 0.3	≤0.001^*∗*^
Omega 6 (mg)	2.1 ± 1.8	15.0 ± 2.4	≤0.001^*∗*^

^*∗*^ *P* ≤ 0.05 is set as significant, SD: standard deviation, RCMD: recommendation, SFA: saturated fatty acid, PUFA: polyunsaturated fatty acid, MUFA: monounsaturated fatty acid, and (mg): milligram.

**Table 10:** Mean intake of amino acids for the participants included in the study.

Amino acids	Nonsarcopenia group mean ± SD	Sarcopenia group mean ± SD	*P* value
Histidine (%)	120.1 ± 38.2	96.0 ± 42.4	0.040^*∗*^
Isoleucine (%)	148.5 ± 42.6	117.8 ± 47.6	0.020^*∗*^
Leucine (%)	117.6 ± 32.8	94.8 ± 37.4	0.026^*∗*^
Lysine (%)	95.2 ± 37.9	76.9 ± 39.6	0.102
Methionine + cysteine (%)	112.4 ± 33.6	96.8 ± 41.3	0.151
Phenylalanine (%)	182.2 ± 206.4	114.6 ± 43.7	0.116
Threonine (%)	115.9 ± 35.6	87.5 ± 35.5	0.007^*∗*^
Tryptophan (%)	147.6 ± 62.5	101.7 ± 39.8	0.003^*∗*^
Valine (%)	139.1 ± 47.3	115.5 ± 45.8	0.080

^*∗*^ *P* ≤ 0.05 is set as significant and SD: standard deviation.

**Table 11:** Mean intake of fat-soluble vitamins for the subjects included in the study.

Vitamins	Sarcopenia group mean ± SD	Control group mean ± SD	*P* value
Vitamin A (IU)	5276.7 ± 4746.4	9276.4 ± 12710.2	0.147
Vitamin A (mcg)	475.2 ± 395.6	699.9 ± 1106.9	0.344
Carotenoid (RE)	399.8 ± 428.3	664.7 ± 1238.3	0.317
Retinol (RE)	290.9 ± 259.1	383.9 ± 934.4	0.634
Beta carotene (mcg)	1870.7 ± 2485.6	3355.3 ± 7292.2	0.340
Vitamin D (IU)	129.5 ± 122.3	93.4 ± 64.5	0.198
Vitamin D (mcg)	3.40 ± 3.1	2.43 ± 1.6	0.177
Vitamin E (mg)	4.28 ± 2.4	5.81 ± 5.2	0.187
Vitamin K (mcg)	108.5 ± 245.8	186.2 ± 241.3	0.265

^*∗*^ *P* ≤ 0.05 is set as significant, SD: standard deviation, IU: international unit, Mcg: microgram, mg: milligram, and RE: retinol equivalent.

**Table 12:** Mean intake of fat-soluble vitamins for the sarcopenic patients compared to their recommendations.

Vitamins	Intake mean ± SD	RCMD mean ± SD	*P* value
Vitamin A (mcg)	475.2 ± 395.2	796.0 ± 101.9	0.001^*∗*^
Vitamin D (mcg)	3.39 ± 3.1	18.8 ± 2.2	≤0.001^*∗*^
Vitamin E (mg)	4.3 ± 2.4	15.0 ± 0.0	≤0.001^*∗*^
Vitamin K (mcg)	108.5 ± 245.8	104.4 ± 15.3	0.935

^*∗*^ *P* ≤ 0.05 is set as significant, SD: standard deviation, Mcg: microgram, mg: milligram, and RCMD: recommendation.

**Table 13:** Mean intake of water-soluble vitamins for the subjects included in the study.

Vitamins	Sarcopenia group mean ± SD	Control group mean ± SD	*P* value
Vitamin B_1_ (mg)	0.86 ± 0.35	0.95 ± 0.56	0.513
Vitamin B_2_ (mg)	1.12 ± 0.44	1.22 ± 0.70	0.567
Vitamin B_3_ (mg)	10.4 ± 5.2	13.9 ± 9.1	0.096
Vitamin B_3_ (niacin equivalent, mg)	15.0 ± 6.9	20.1 ± 11.0	0.058
Vitamin B_6_ (mg)	0.88 ± 0.49	1.08 ± 0.51	0.163
Vitamin B_12_ (mcg)	2.99 ± 1.5	3.82 ± 10.1	0.683
Biotin (mcg)	11.3 ± 3.2	12.4 ± 5.1	0.350
Folate (mg)	251.5 ± 137.0	258.7 ± 121.9	0.845
Dietary folate equivalent (DFE) (mcg)	329.7 ± 163.6	283.2 ± 162.8	0.319
Pantothenic acid (mg)	3.61 ± 1.6	3.47 ± 1.6	0.767
Vitamin C (mg)	65.88 ± 78.9	143.5 ± 79.6	0.001^*∗*^

^*∗*^ *P* ≤ 0.05 is set as significant, SD: standard deviation, DFE: dietary folate equivalent, mcg: microgram, and mg: milligram.

**Table 14:** Mean intake of water-soluble vitamins for the sarcopenic patients compared to their recommendations.

Vitamins	Intake mean ± SD	RCMD mean ± SD	*P* value
Vitamin B_1_ (mg)	0.86 ± 0.35	1.15 ± 0.05	0.001^*∗*^
Vitamin B_2_ (mg)	1.12 ± 0.4	1.2 ± 0.1	0.466
Vitamin B_3_ (mg)	14.96 ± 1.0	14.99 ± 6.9	≤0.001^*∗*^
VitaminB_3_ (niacin equivalent,mg)	14.99 ± 6.9	14.96 ± 1.0	0.982
Vitamin B_6_ (mg)	0.88 ± 0.5	1.58 ± 0.1	≤0.001^*∗*^
Vitamin B_12_ (mcg)	2.99 ± 1.5	2.4 ± 0.0	0.056
Biotin (mcg)	11.3 ± 3.1	30 ± 0.0	≤0.001^*∗*^
Folate (mg)	251.5 ± 137.0	400 ± 0.0	≤0.001^*∗*^
Dietary folate equivalent (DFE) (mcg)	3.61 ± 1.6	5.0 ± 0.0	≤0.001^*∗*^
Pantothenic acid (mg)	65.9 ± 78.9	82.2 ± 7.6	0.322

^*∗*^ *P* ≤ 0.05 is set as significant, SD: standard deviation, RCMD: recommendation, (mcg): microgram, and (mg): milligram.

**Table 15:** Mean intake of major and trace minerals for the participants included in the study.

Minerals	Sarcopenia group mean ± SD	Nonsarcopenia group mean ± SD	*P* value
Calcium (mg)	662.1 ± 268.1	776.6 ± 329.3	0.184
Chromium (mcg)	2.04 ± 1.5	2.07 ± 1.4	0.936
Copper (mg)	0.63 ± 0.4	1.45 ± 1.9	0.047^*∗*^
Fluoride (mg)	0.37 ± 0.4	1.41 ± 0.6	≤0.001^*∗*^
Iodine (mcg)	90.2 ± 34.2	60.7 ± 36.1	0.005^*∗*^
Iron (mg)	9.22 ± 3.8	11.86 ± 8.5	0.164
Magnesium (mg)	165.8 ± 82.2	248.4 ± 115.7	0.005^*∗*^
Manganese (mg)	1.71 ± 1.1	4.01 ± 2.3	≤0.001^*∗*^
Molybdenum (mcg)	23.6 ± 34.1	22.4 ± 29.6	0.899
Phosphorus (mg)	712.2 ± 214.4	783.2 ± 236.5	0.272
Potassium (mg)	1476.9 ± 526.9	2118.5 ± 649.0	≤0.001^*∗*^
Selenium (mcg)	50.95 ± 29.0	44.43 ± 23.7	0.389
Sodium (mg)	1962.3 ± 3553.6	2308.3 ± 1339.3	0.651
Zinc (mg)	6.19 ± 2.5	6.14 ± 2.7	0.946

^*∗*^ *P* ≤ 0.05 is set as significant, (mcg): microgram, (mg): milligram, and SD: standard deviation.

**Table 16:** Mean intake of minerals for the sarcopenic patients compared to their recommendations.

Minerals	Intake mean ± SD	RCMD mean ± SD	*P* value
Calcium (mg)	662.1 ± 268.1	1168.0 ± 74.8	≤0.001^*∗*^
Chromium (mcg)	2.04 ± 1.4	25.2 ± 4.9	≤0.001^*∗*^
Copper (mg)	0.63 ± 0.39	0.90 ± 0.0	0.003^*∗*^
Fluoride (mg)	0.37 ± 0.43	3.5 ± 0.5	≤0.001^*∗*^
Iodine (mcg)	90.2 ± 34.2	150 ± 0.0	≤0.001^*∗*^
Iron (mg)	9.2 ± 3.8	8.8 ± 2.8	0.613
Magnesium (mg)	165.8 ± 82.1	376.6 ± 51.4	≤0.001^*∗*^
Manganese (mg)	1.7 ± 1.1	2.0 ± 0.3	0.176
Molybdenum (mcg)	23.6 ± 34.1	45.0 ± 0.0	0.004^*∗*^
Phosphorus (mg)	712.2 ± 214.4	700 ± 0.0	0.778
Potassium (mg)	1476.9 ± 526.8	4700 ± 0.0	≤0.001^*∗*^
Selenium (mcg)	50.9 ± 29.0	55 ± 0.0	0.493
Sodium (mg)	1962.3 ± 3553.6	2300 ± 0.0	0.639
Zinc (mg)	6.19 ± 2.5	9.44 ± 1.5	≤0.001^*∗*^

^*∗*^ *P* ≤ 0.05 is set as significant, (mcg): microgram, (mg): milligram, and SD: standard deviation.

**Table 17:** Nutritional assessment for participants included in the study.

Nutritional assessment	Sarcopenia group *N* (%)	Nonsarcopenia group *N* (%)	*P* value
*Can you manage eating without any help?*			
Dependent	3 (12)	0 (0)	
Verbal assistance	0 (0)	0 (0)	≤0.001^*∗*^
Some human help	13 (52)	0 (0)	
Independent	9 (36)	25 (100)	

*Can you prepare meals for yourself without help?*			
Dependent	21 (84)	0 (0)	
Verbal assistance	0 (0)	0 (0)	≤0.001^*∗*^
Some human help	2 (8)	1 (4)	
Independent	2 (8)	24 (96)	

*Can you shop for food and other things you need without help?*			
Dependent	22 (88)	1 (4)	≤0.001^*∗*^
Verbal assistance	0 (0)	0 (0)	
Some human help	1 (4.0)	2 (8.0)	
Independent	2 (8)	22 (88)	

*Can you pick up the food?*			
Dependent	3 (12)	0 (0)	
Verbal assistance	0 (0)	0 (0)	0.001^*∗*^
Some human help	8 (32)	0 (0)	
Independent	14 (55)	25 (100)	

*Can you grasp utensils and cups?*			
Dependent	5 (20)	0 (0)	
Verbal assistance	0 (0)	0 (0)	≤0.001^*∗*^
Some human help	16 (64)	1 (4)	
Independent	4 (16)	24 (96)	

*Can you get food on utensils?*			
Dependent	1 (4.0)	0 (0)	
Verbal assistance	0 (0)	0 (0)	≤0.001^*∗*^
Some human help	14 (56)	0 (0)	
Independent	10 (40)	25 (100)	

*Can you bring food, utensils, cups to mouth?*			
Dependent	1 (4)	0 (0)	
Verbal assistance	0 (0)	0 (0)	0.017^*∗*^
Some human help	6 (24)	0 (0)	
Independent	18 (72)	25 (100)	

*Can you chew, swallow food and liquids?*			
Dependent	5 (20)	0 (0)	
Verbal assistance	0 (0)	0 (0)	≤0.001^*∗*^
Some human help	8 (32)	0(0)	
Independent	12 (48)	25 (100)	

*Can you clean your mouth and hands as necessary following a meal?*			
Dependent	0 (0)	0 (0)	
Verbal assistance	0 (0)	0 (0)	≤0.001^*∗*^
Some human help	10 (40)	0(0)	
Independent	15 (60)	25 (100)	

*Is there any weight loss during the last 3 months?*			
No	8 (32)	6 (24)	
Does not know	3 (12)	11 (44)	0.039^*∗*^
Between 1 and 3 kg	14 (56)	8 (32)	
>3 kg	0 (0)	0 (0)	

*Has food intake declined over the past 3 months due to loss of appetite, digestive problems, chewing or swallowing difficulties?*			
Severe loss of appetite	0 (0)	0 (0)	
Moderate loss of appetite	12 (48)	1 (4)	≤0.001^*∗*^
No loss	13 (52)	24 (96)	

*How many full meals do you eat daily?*			
1 meal	0 (0)	1 (4)	
2 meals	10 (40)	21 (84)	0.002^*∗*^
3 meals	15 (60)	3 (12)	
>3 meals	0 (0)	0 (0)	

*How many snacks do you eat daily?*			
1 meal	14 (56)	10 (40)	
2 meals	11 (44)	13 (52)	0.243
3 meals	0 (0)	2 (8)	
>3 meals	0 (0)	0 (0)	

*How much vegetables being consumed per day?*			
One	10 (40)	0 (0)	
Two	14 (56)	5 (20)	≤0.001^*∗*^
Three	1 (4)	15 (60)	
> Three	0 (0)	5 (20)	

*How much fruits being consumed per day?*			
One	23 (92)	16 (64)	
Two	2 (8)	9 (36)	0.017^*∗*^
Three	0 (0)	0 (0)	
> Three	0 (0)	0 (0)	

*How much water is consumed per day?*			
<3 cups	1 (4)	0 (0)	
5 cups	12 (48)	0 (0)	≤0.001^*∗*^
7 cups	8 (32)	14 (56)	
>7 cups	4 (16)	11 (44)	

*How much juice is consumed per day?*			
None	6 (24)	8 (32)	
One cup	18 (72)	16 (64)	0.817
2 cups	1 (4)	1 (4)	
>2 cups	0 (0)	0 (0)	

*How much milk is consumed per day?*			
None	5 (20)	4 (16)	0.933
One cup	17 (68)	18 (72)	
2 cups	3 (12)	3 (12)	
>2 cups	0 (0)	0 (0)	

*How much tea and coffee consumed per day?*			
None	7 (28)	0 (0)	≤0.001^*∗*^
One cup	12 (48)	3 (12)	
2 cups	6 (24)	14 (56)	
>2 cups	0(0)	8 (32)	

*How much white or brown bread consumed per day?*			
None	1 (4.0)	0 (0)	
<Loaf	18 (72)	11 (44)	0.053
1–2 loaf	6 (24)	14 (56)	
>2 loaf	0 (0)	0 (0)	

*How much rice or spaghetti consumed per day?*			
None	0 (0)	0 (0)	
One cup	17 (68)	6 (24)	0.002^*∗*^
2 cups	8 (32)	19 (76)	
>2 cups	0 (0)	0 (0)	

*How many serving of dairy products did you eat daily?*			
None	0 (0)	0 (0)	
1–2 serving	24 (96)	22 (88)	0.297
3–4 serving	1 (4)	3 (12)	
>4 serving	0 (0)	0 (0)	

*How much meat or chicken or fish consumed per day?*			
None	0 (0)	0 (0)	
<1 piece	13 (52)	0 (0)	≤0.001^*∗*^
1–2 piece	12 (48)	25 (100)	
>2 serving	0 (0)	0 (0)	

*How often do you eat eggs?*			
None	1 (4)	0 (0)	
One egg	24 (96)	18 (72)	0.012^*∗*^
2–3 eggs	0 (0)	7 (28)	
>3 eggs	0 (0)	0 (0)	

*How often do you eat nuts like pistachios, cashews, hazelnuts, etc.?*			
None	18 (72)	4 (16)	
Once a day	7 (28)	14 (56)	≤0.001^*∗*^
Twice a day	0 (0)	7 (28)	
>2 a day	0 (0)	0 (0)	

*N* represents the number of participants, (%) represents the percentage of the participants, and ^*∗*^*P* ≤ 0.05 is set as significant.

**Table 18:** Nutritional risk assessment for the participants included in the study.

Assessment	Nonsarcopenia group *N* (%)	Sarcopenia group *N* (%)	*P* value
High nutritional risk	0 (0)	2 (8)	≤0.001^*∗*^
Moderate nutritional risk	1 (4)	17 (68)	
No nutritional risk	24 (96)	6 (24)	

*N* represents the number of participants, (%) represents the percentage of the participants, and ^*∗*^*P* ≤ 0.05 is set as significant.

**Table 19:** Physical activity for participants included in the study.

Physical activity	Sarcopenia group *N* (%)	Control group *N* (%)	*P* value
*Over the past 7 days, how often did you take a walk outside your home or yard for any reason? For example, for fun or exercise, walking to work, etc.?*			
Never	9 (36)	1 (4)	
Seldom (1–2 days)	13 (52)	1 (4)	≤0.001^*∗*^
Sometimes (3–4 days)	3 (12)	15 (60)	
Often (5–7 days)	0 (0)	8 (32)	

*Over the past 7 days, how often did you engage in light sport or recreational activities?*			
Never	23 (92)	17 (68)	
Seldom (1–2 days)	1 (4)	8 (32)	0.025^*∗*^
Sometimes (3–4 days)	1 (4)	0 (0)	
Often (5–7 days)	0 (0)	0 (0)	

*Over the past 7 days, how often did you engage in moderate sport and recreational activities?*			
Never	25 (100)	25 (100)	
Seldom (1–2 days)	0 (0)	0 (0)	
Sometimes (3–4 days)	0 (0)	0 (0)	
Often (5–7 days)	0 (0)	0 (0)	

*Over the past 7 days, how often did you engage in strenuous sport and recreational activities?*			
Never	25 (100)	25 (100)	
Seldom (1–2 days)	0 (0)	0 (0)	
Sometimes (3–4 days)	0 (0)	0 (0)	
Often (5–7 days)	0 (0)	0 (0)	

*Over the past 7 days, how often did you do any exercises specifically to increase muscle strength and endurance, such as lifting weights or push-ups, etc.?*			
Never	25 (100)	15 (60)	
Seldom (1–2 days)	0 (0)	5 (20)	0.006^*∗*^
Sometimes (3–4 days)	0 (0)	4 (16)	
Often (5–7 days)	0 (0)	1 (4)	

*During the past 7 days, have you done any light housework, such as dusting or washing dishes?*			
Never	5 (20)	2 (8)	
Seldom (1–2 days)	16 (64)	8 (32)	0.001^*∗*^
Sometimes (3–4 days)	4 (16)	2 (8)	
Often (5–7 days)	0(0)	13 (52)	

*N* represents the number of participants, (%) represents the percentage of the participants, and ^*∗*^*P* ≤ 0.05 is set as significant.

**Table 20:** The physical activity level for the subjects included in the study.

Physical activity level	Sarcopenia group N (%)	Control group N (%)	*P* value
Highly active	0 (0)	0 (0)	≤0.001^*∗*^
Moderately active	0 (0)	6 (24)	
Lightly active	7 (28)	18 (72)	
Sedentary activity	18 (72)	1(4)	

*N* represents the number of participants, (%) represents the percentage of the participants, and ^*∗*^*P* ≤ 0.05 is set as significant.

**Table 21:** Nutritional assessment form.

1. Can you manage eating without any help?	(A) Independent	(B) Verbal assistance	(C) Some human help	(D) Dependent
2. Can you prepare meals for yourself without help?	(A) Independent	(B) Verbal assistance	(C) Some human help	(D) Dependent
3. Can you shop for food and other things you need without help?	(A) Independent	(B) Verbal assistance	(C) Some human help	(D) Dependent
4. Can you pick up the food?	(A) Independent	(B) Verbal assistance	(C) Some human help	(D) Dependent
5. Can you grasp utensils and cups?	(A) Independent	(B) Verbal assistance	(C) Some human help	(D) Dependent
6. Can you get food on utensils?	(A) Independent	(B) Verbal assistance	(C) Some human help	(D) Dependent
7. Can you bring food, utensils, cups to mouth?	(A) Independent	(B) Verbal assistance	(C) Some human help	(D) Dependent
8. Can you chew, swallow food and liquids?	(A) Independent	(B) Verbal assistance	(C) Some human help	(D) Dependent
9. Can you clean your mouth and hands as necessary following a meal?	(A) Independent	(B) Verbal assistance	(C) Some human help	(D) Dependent
10. Is there any weight loss during the last 3 months?	(A) No weight loss	(B) Does not know	(C) Between 1 and 3 kg	(D) >3 kg
11. Has food intake declined over the past 3 months due to loss of appetite, digestive problems, chewing or swallowing difficulties?	(A) Severe loss of appetite	(B) Moderate loss of appetite	(C) No loss	
12. How many full meals do you eat daily?	(A) 1meal	(B) 2 meals	(C)3 meals	(D) >3 meals
13. How many snacks do you eat daily?	(A) 1 meal	(B) 2 meals	(C)3 meals	(D) >3 meals
14. How much vegetables being consumed per day?	(A) One	(B) Two	(C) Three	(D) >three
15. How much fruits being consumed per day?	(A) One	(B) Two	(C) Three	(D) >three
16. How much water is consumed per day?	(A) <3 cups	(B) 5 cups	(C) 7 cups	(D) >7 cups
17. How much juice is consumed per day?	(A) None	(B) One cup	(C) 2 cups	(D) >2 cups
18. How much milk is consumed per day?	(A) None	(B) One cup	(C) 2 cups	(D) >2 cups
19. How much tea and coffee consumed per day?	(A) None	(B) One cup	(C) 2 cups	(D) >2 cups
20. How much white or brown bread consumed per day?	(A) None	(B) <loaf	(C) 1-2 loaf	(D) >2 loaf
21. How much rice or spaghetti consumed per day?	(A) None	(B) <cup	(C) 1-2 cup	(D) >2 cups
22. How many serving of dairy products did you eat daily?	(A) None	(B) 1–2 serving	(C) 3–4 serving	(D) >4 servings
23. How much meat or chicken or fish consumed per day?	(A) None	(B) <1 piece	(C) 1–2 piece	(D) >2 serving
24. How often do you eat eggs?	(A) None	(B) One egg	(C) 2–3 eggs	(D) >3 eggs
25. How often do you eat nuts like pistachios, nuts, cashews, hazelnuts, etc.?	(A) None	(B) once a day	(C) Twice a day	(D) >2 a day

## Data Availability

The data used to support the findings of this study are included within the article.
